# Clinical and Histopathologic Predictors of Survival Among Children With Retinoblastoma From Two Tertiary Health Facilities in Uganda

**DOI:** 10.7759/cureus.50605

**Published:** 2023-12-15

**Authors:** Raymond Atwine, Damaris Laffita, Abraham Birungi, Ritah Kiconco, Keith Waddell

**Affiliations:** 1 Pathology, Mbarara University of Science and Technology, Mbarara, UGA; 2 Pathology, Angel Arturo Aballi Hospital, Havana, CUB; 3 Biochemistry, Soroti University, Soroti, UGA; 4 Medical Laboratory Science, Mbarara University of Science and Technology, Mbarara, UGA; 5 Ophthalmology, Ruharo Eye Centre, Mbarara, UGA

**Keywords:** children, survival, retinoblastoma, features, pathological, histology, clinical features, predictors, uganda

## Abstract

Background: Retinoblastoma (RB) is a malignant tumour that develops from the immature cells of the retina. It is the most frequent type of paediatric intraocular cancer and is curable. Clinical and histological findings after enucleation of the affected eye dictate not only the patient's secondary care but also their prognosis. We assessed the clinical and histopathologic predictors of survival among children with RB from two tertiary health facilities in Uganda.

Methods: This retrospective research utilized archived formalin fixed and paraffin-embedded blocks of eye specimens enucleated between 2014 and 2016 at Mbarara University of Science and Technology (MUST) Pathology Department and Ruharo Eye Centre (REC) in Mbarara, Uganda. The specimens were then processed and stained with haematoxylin and eosin. The confirmation of RB was made to include the histologic stage and features of the tumor. Biographic data of the patients and clinical features, such as leukocoria, proptosis, phthisis, staphyloma and buphthalmos, were retrieved from the records.

Results: Males (55.1%, n=43) dominated the study population (N=78). The median age was 31 months. The most common clinical sign was leukocoria (69.2%, n=52), and the most predominant histopathological stage was stage 1 (41%, n=32). Optic nerve (ON) invasion was seen in 38.5% (n=30), choroidal invasion in 29.5% (n=23), scleral invasion in 7.7% (n=6) and orbital extension in 16.7% (n=13) of the cases. Flexner-Wintersteiner rosettes were seen in 34.6% (n=27). Necrosis was a prominent feature (71.8%, n=56). The two-year survival was estimated to be 61.5% (n=48). Leukocoria (risk ratio (RR) 1.1), female gender (RR 1.4), intralaminar ON invasion (RR 7.6) and a lack of orbital extension (RR 7) were significant predictors of survival.

Conclusion: Leukocoria and proptosis are noticeable clinical signs of RB. Most patients present while in stage one although stage four presentation is also common. Leukocoria, ON invasion, orbital extension and gender are significant factors predictive of survival in patients with RB.

## Introduction

Retinoblastoma (RB) is a malignant tumor that develops from the immature cells of the retina [1]. It is the most common intraocular cancer of childhood affecting approximately 1 in 15,000-20,000 births, with an incidence of 7000-8000 new cases worldwide and 4000 deaths annually [2]. It is a common cause of blindness, morbidity and mortality particularly in the underdeveloped countries of sub-Saharan Africa [3]. Regions with the greatest prevalence have the highest mortality with up to 70% mortality in Asia and Africa, compared with 3-5% in Europe, Canada and the USA [4]. In Uganda, RB is the fifth most common cancer after lymphomas, Kaposi sarcoma, leukemia and nephroblastoma [5].

The survival of RB patients in Africa, Uganda in particular, is low largely because of delayed presentation [6]. The advanced stage of disease is found to be associated with very poor outcomes. Survival majorly depends on the severity of disease at presentation. Survival rates in the UK and USA approach 100% with survival in other countries, primarily developing nations, much lower. Survival rates have been reported to be 80-89% in developed Latin American countries, 48% in India and as low as 20-46% in Africa. In Uganda, survival rose from 45% in the pre-chemotherapy era to 65% in the post chemotherapy era [6].

A worldwide issue is poor access to comprehensive RB pathology [4]. Histological examination of the enucleated globes in the region has also been inconsistent, as shown in studies done in Uganda and Kenya [7,8]. RB management not informed by histological examination could impede development of a rational management plan and lead to unsatisfactory clinical outcomes.

There is a paucity of data on the association between clinical and histopathological features and survival so as to guide appropriate RB management and ultimately improve survival. This study intends to address this gap.

## Materials and methods

Study design and site

This was a retrospective study carried out at two health facilities in southwestern Uganda. Mbarara Regional Referral Hospital (MRRH) is a tertiary hospital with a 350-bed capacity, which is government-funded through the Ministry of Health intended to provide free service and covers the districts of the southwestern region of the country. It also receives patients from the neighboring nations of the Democratic Republic of Congo (DRC), Rwanda and Tanzania. It is a teaching hospital for Mbarara University of Science and Technology (MUST) and other health training institutions in the region.

Another study site was Ruharo Eye Centre (REC). REC is one of the referral centers for RB cases in the country and receives over 90 cases of RB annually.

The enucleated specimens were taken to MUST histopathology laboratory in the MUST pathology department, which is the only government-aided histopathology laboratory in the southern and western parts of Uganda. The MUST pathology department is a referral unit for cases that require histologic diagnosis in the region.

Study variables

Clinical features were retrieved from archived records. These included leukocoria, strabismus, proptosis, uveitis, cataract, staphyloma, phthisis, laterality and treatment. Clinical outcome data were recorded as either alive or dead. The staging was performed using the American Joint Committee on Cancer (AJCC) 8th tumor, node, metastasis (TNM8) staging system staging system as pT1, PT2, pT3 and pT4.

The following histologic features were noted: growth patterns as exophytic, endophytic and mixed; invasion of lens, conjunctiva and corneal epithelium; invasion of anterior segment structures as present or absent (iris, ciliary body and trabecular meshwork); necrosis as none, mild (involving less than 25%), moderate (25-50%) and extensive (more than 50%); calcification as none, mild (involving less than 25%), moderate (25-50%) and extensive (more than 50%); and Flexner-Wintersteiner rosettes as mild (0-25%), moderate (25- 50%) and many (more than 50%).

The well-differentiated tumors were those constituting more than 50% of the rosettes, moderately differentiated as those less than 50% rosettes and poorly differentiated as those without any rosettes; Homer-Wright rosettes as absent or present; mitosis as present or absent; and presence of inflammation as chronic (lymphohistiocytic) or acute inflammation.

Data analysis

Stata Statistical Software release 13 (2013, StataCorp. LLC, College Station, Texas, USA) was used for the analysis. Baseline participants’ characteristics were described using appropriate summary statistics, which are the mean or median for continuous variables and proportions for categorical variables. The histopathological stages of RB among children were also presented as proportions. Survival after two years in care was computed as a cumulative measure and expressed as a proportion of all children still alive by two years out of all that were admitted with RB at REC. The corresponding 95% confidence interval (CI) was also reported.

Independent variables included sociodemographic factors, such as age, gender and geographical region of residence, in-hospital care, histopathologic features of the tumor and clinical presentation of the children. Unadjusted risk ratios (RRs) were reported together with their corresponding 95% CI. A significance level of 5% was used. All independent variables with p<0.1 were included in the multivariate model building using a manual backward-stepwise selection method. Variables that lost their association with survival at two years were excluded from the final multivariate model. In addition, variables that could not allow for the convergence of the model were excluded. For all variables in the final model, adjusted RRs were presented with their corresponding 95% CIs.

## Results

We included 78 eye specimens in the study. As shown in Table 1, the median age of diagnosis was 31 months, and most of the participants were between 12 and 59 months (78.4%, n=58). Majority were males (55.1%, n=43), and most of them originated from western Uganda (33.3%, n=26). In most cases, only one eye was affected at the time of diagnosis (70.5%, n=55).

**Table 1:** Baseline characteristics of the sample source patients

Characteristic	n(%)
Age in months, median (interquartile range (IQR))	31 (18-39)
Age categories (months)	
<12	10 (12.8)
12-59	58 (78.4)
≥60	10 (12.8)
Gender	
Male	43 (55.1)
Female	35 (44.9)
Region	
Western	26 (33.3)
Eastern	21 (26.9)
Central	22 (28.2)
Northern	5 (6.4)
International	4 (5.1)
Laterality	
Unilateral	55 (70.5)
Bilateral	23 (29.5)
Chemo-reduction therapy	
Given	63 (80.8)
Not given	15 (19.2)

**Figure 1:**
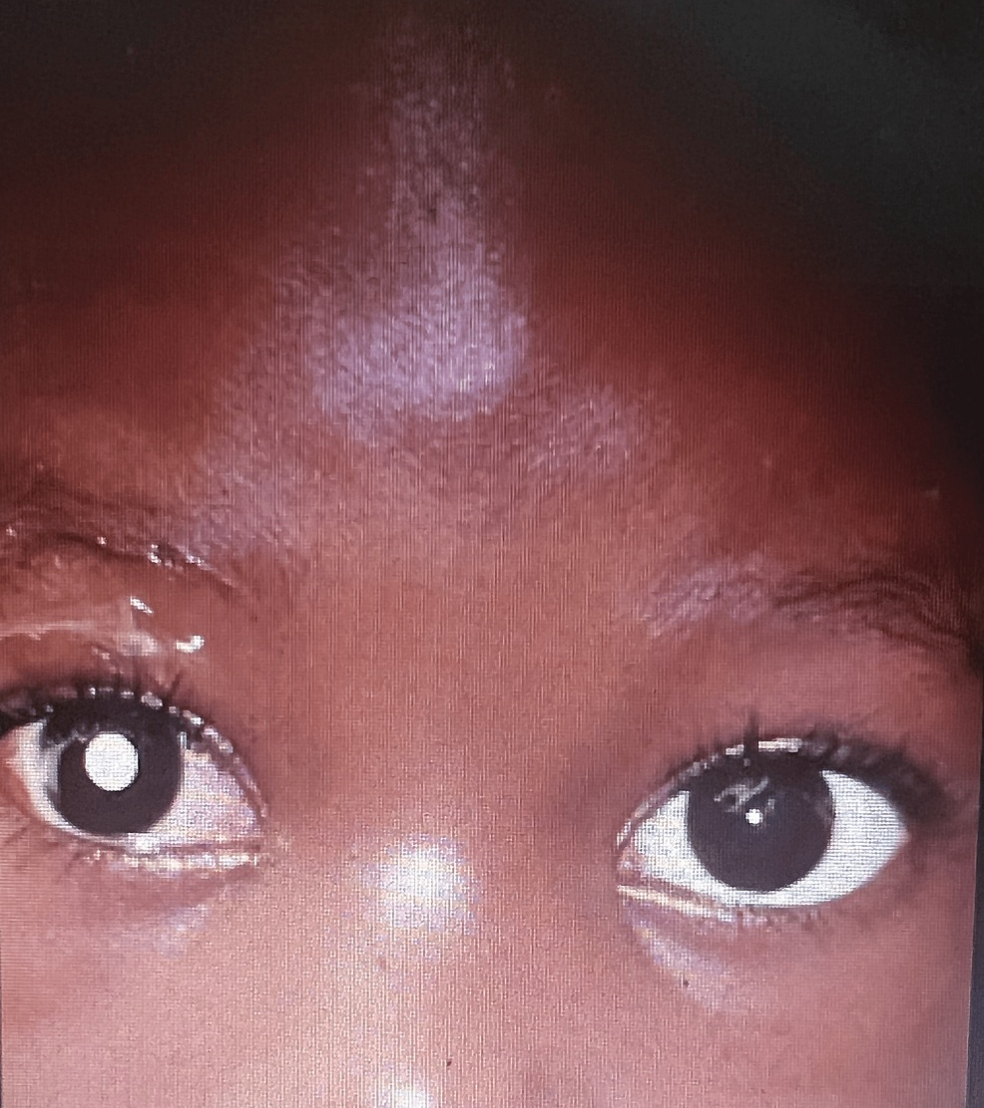
Image showing a patient with leukocoria

**Figure 2:**
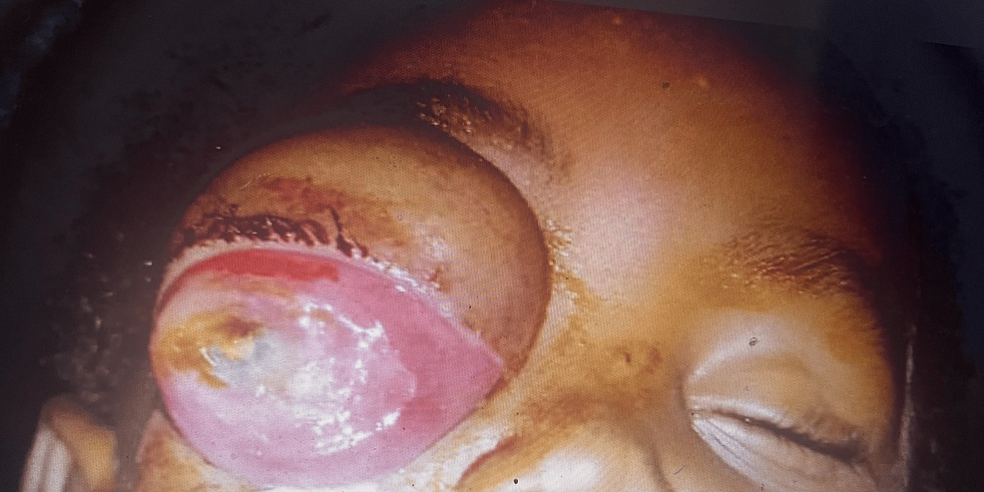
Image showing a patient with proptosis

The most common clinical sign was leukocoria (69.2%, n=52), which was followed by proptosis (32.1%, n=25), uveitis (16.7%, n=13), phthisis (12.8%, n=10), buphthalmos (9%, n=7), staphyloma (5.1%, n=4), cataracts (1.3%, n=1) and lastly strabismus (1.3%, n=1). The most common pathologic stage was stage 1 (41.0%, n=32), followed by stage 4 (26.9%, n=21), stage 2 (26.9%, n=21) and lastly stage 3 (5.1%, n=4) (Figures 1, 2).

**Figure 3:**
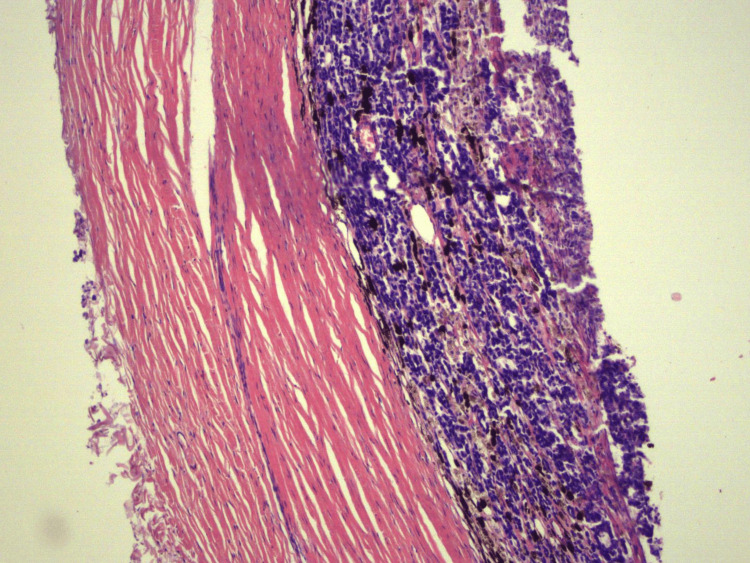
Massive choroidal invasion x200

**Figure 4:**
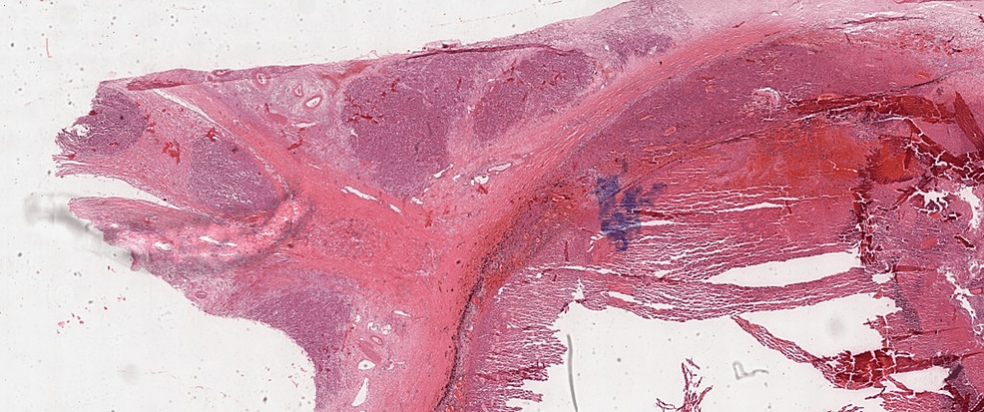
Optic nerve and extra scleral (orbital) invasion x20

**Figure 5:**
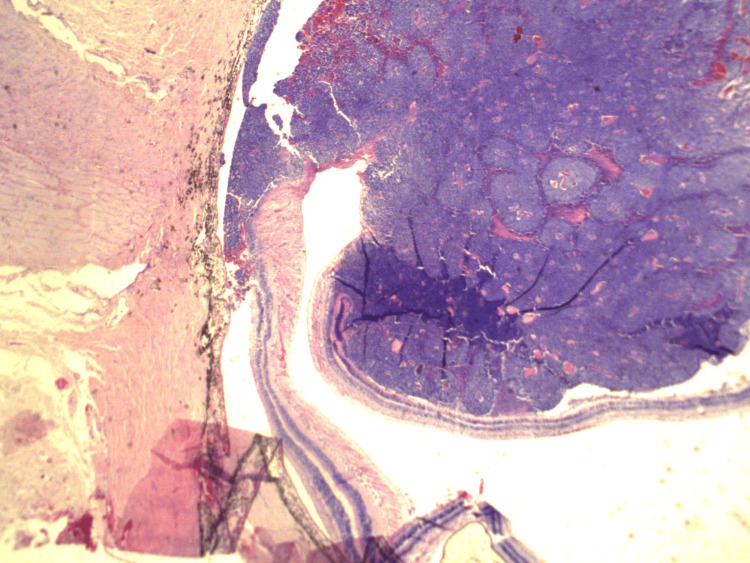
Endophytic tumor x100

Choroidal invasion was seen in 29.5% (n=23) of the specimens, and more than half of these were massive (Figure 3). Optic nerve (ON) invasion was seen in 38.5% (n=30) cases (Figure 4), with almost half of these having invasion to the surgical end/margin. Orbital extension was seen in 16.7% (n=13) cases, while scleral invasion was seen in a paltry 7.7% (n=6). Iris, trabecular meshwork and ciliary body invasion accounted for 16.7% (n=13) each. Lens, corneal, conjunctival and vascular invasion accounted for a combined paltry 7.7% (n=6). Endophytic tumor was seen in 71.8% (n=56) cases (Figure 5). Flexner-Wintersteiner rosettes were seen in 34.6% (n=27) cases, while Homer-Wright rosettes in only 6.4% (n=5) cases. Necrosis was seen in 71.8% (n=56) cases, calcification was seen in 41% (n=32) cases, and mitoses were seen in only 9% (n=7) cases (Table 2).

**Table 2:** Histopathologic features of the samples

Characteristic	n(%)
Choroidal invasion	
Absent	55 (70.5)
Focal	13 (16.7)
Massive	10(12.8)
Optic nerve invasion	
Absent	48 (61.5)
Prelaminar	10 (12.8)
Intra-laminar	4 (5.1)
Retrolaminar	3 (3.9)
Surgical margin/cut end	13 (16.7)
Scleral invasion	
Absent	72 (92.3)
Partial thickness	1 (1.3)
Full thickness	5 (6.4)
Orbital extension	13 (16.7)
Iris	13 (16.7)
Trabecular meshwork	13 (16.7)
Growth pattern	
Endophytic	56 (71.8)
Exophytic	14 (17.9)
Mixed	8 (10.3)
Lens Invasion	1 (1.3)
Ciliary body invasion	13 (16.7)
Corneal epithelium invasion	2(2.6)
Conjunctival invasion	1 (1.3)
Vascular invasion	2(2.6)
Flexner-Wintersteiner rosettes	
None	51 (65.4)
Mild	12 (15.4)
Moderate	7 (9.0)
Many	8 (10.2)
Homer-Wright rosettes	
None	73 (93.6)
Present	5 (6.4)
Necrosis	
None	22 (28.2)
Mild	18 (23.1)
Moderate	12 (15.4)
Massive	26 (33.3)
Calcification	
None	46 (59.0)
Mild	13 (16.6)
Moderate	12 (15.4)
Massive	7 (9.0)

The two-year survival was estimated to be 61.5% (n=48), as shown in Table 3.

**Table 3:** Results of univariate analysis showing predictors of two-year survival

Characteristic	Dead n (%)	Alive n (%)	Unadjusted risk ratio (RR) (95% CI)	p value
Gender				
Female	7 (20)	28 (80)	1.7 (1.20- 2.47)	0.003
Male	23 (53.5)	20 (46.5)	1.0	
Region				
Eastern	11 (52.4)	10 (47.6)	0.8 (0.45- 1.33)	0.354
Central	7 (61.8)	15 (38.2)	1.1 (0.73- 1.68)	0.630
Southern	0 (0)	5 (100)	1.7 (1.26- 2.30)	0.001
International	2 (50)	2 (50)	0.8 (0.29- 2.27)	0.692
Western	10 (38.5)	16 (61.5)	1.0	
Leukocoria				
Present	15 (27.8)	39 (72.2)	1.9 (1.12- 3.31)	0.018
Absent	15 (62.5)	9 (37.5)	1.0	
Proptosis				
Present	20 (80)	5 (20)	4.1 (1.83- 8.98)	≤ 0.001
Absent	10 (18.9)	43 (81.1)	1.0	
Cataract				
Present	0 (0)	1 (100)	1.7(1.43- 2.05)	≤ 0.001
Absent	30 (38)	47 (61)	1.0	
Choroidal invasion				
Absent	17 (30.9)	38 (69.1)	6.9 (1.07- 44.72)	0.043
Focal	4 (30.8)	9 (69.2)	6.9 (1.04- 46.03)	0.045
Massive	9 (90)	1 (10)	1.0	
Optic nerve invasion				
Absent	14 (29.2)	34 (70.8)	9.2 (1.39- 61.06)	0.021
Prelaminar	2 (20)	8 (80)	10.4 (1.4- 70.12)	0.016
Intralaminar	0 (0)	4 (100)	13.6 (2.07- 89.4)	0.007
Retrolaminar	2 (66.7)	1 (33.3)	4.3 (0.37- 51.29)	0.245
Surgical margin/cut end	12 (92.3)	1 (7.7)	1.0	
Orbital extension				
Absent	18 (27.7)	47 (72.3)	9.4 (1.42- 62.16)	0.020
Present	12 (92.3)	1 (7.7)	1.0	
Necrosis				
None	3 (13.6)	19 (83.4)	2.3 (1.34- 2.75)	0.002
Mild	8 (44.4)	10 (55.6)	1.4 (0.76- 2.73)	0.259
Moderate	3 (25)	9 (75)	1.95 (1.08- 3.50)	0.025
Severe	16 (61.5)	10 (38.5)	1.0	

At univariate analysis, gender, region of origin, leukocoria, proptosis, cataract, choroidal invasion, orbital invasion and necrosis were significant factors in predicting survival. Age, laterality, chemoreduction, buphthalmos, growth pattern, calcification, vascularity, mitosis and differentiation were not significant. Scleral invasion and anterior segment invasion were not able to produce an RR because the alive category had zeroes (Table 4).

**Table 4:** Estimation of survival at two years.

Status at 2 years	n (%)	Proportion (confidence interval (CI))
Dead	30 (38.5)	38 (28- 50)
Alive	48 (61.5)	62 (50- 72)

Female gender, leukocoria, proptosis, choroidal invasion, ON invasion orbital invasion, region, cataract and necrosis were run in the final model; however, region, cataract, choroidal invasion, proptosis and necrosis would not bring convergence, so they were eliminated from the final model.

Female gender, leukocoria, orbital extension and ON invasion were significant predictors of survival, with females being able to survive 1.4 times better than males and patients without leukocoria being able to survive 1.1 times better than those without.

Patients without ON invasion will survive better than those with ON invasion depending on the degree of invasion, and patients without orbital extension will be able to survive seven times better than those with orbital extension (Table 5).

**Table 5:** Predictors of two-year survival based on the multivariate analysis

Variable	Adjusted risk ratio (RR) (95% confidence interval (CI))	P value
Gender		
Female	1.4 (1.07-1.70)	0.009
Male	1.0	
Leukocoria		
Present	(1.10-1.11)	≤0.001
Absent	1.0	
Optic nerve (ON) invasion		
Absent	6.0 (0.85-42.3)	0.072
Prelaminar ON invasion	7.0 (0.99-49.27)	0.052
Intralaminar ON invasion	7.6 (1.08-53.98)	0.042
Retrolaminar ON invasion	2.4 (0.18-31.04)	0.504
Surgical margin ON invasion	1.0	
Orbital extension		
Absent	7.0 (1.00-49.25)	0.002
Present	1.0	

## Discussion

The median age of the patients at presentation was 31 months, which is comparable to a study at a tertiary center in Kinshasa, Democratic Republic of Congo, that found the median age at 32 months and 29 months in an Indian population. However, this is not comparable to studies done in developed countries that reported a median age of 12 months, such as in the UK. This is attributed to the late presentation and delayed diagnosis of these cases in our setting and other developing countries [9].

The prevalence of males and females varies widely from studies. Males accounted for 55.1% (n=43), which was comparable to a Kenyan study with 54% and other studies in both developed and developing countries, such as Turkey and Pakistan [8]. However, studies done in Malaysia and Nigeria have reported a higher prevalence of females. This could be attributed to the genetic differences in the different populations and referral selection due to differences in cultural beliefs.

The most common age groups involved was the 12-59 months (78.4%, n=58). Delayed diagnosis is commonly encountered in developing countries with 90% of cases diagnosed before the age of five years as evidenced by a study done in Cameroon, which is consistent with our study at 91.2% and 85% in Nigeria [10]. On the contrary, most children diagnosed with RB in developed countries are less than 24 months old because of the early presentation and diagnosis [1]. The delayed diagnosis in developing countries is due to the lack of awareness and poor accessibility to referral/tertiary centers where these patients can be ably managed [9].

Unilateral cases were 70.5% (n=55), and this is comparable to many studies that reported unilateral cases to be 72%, 74% and 71.2% in Kenya, Uganda and Pakistan, respectively [7,8]. Sub-Saharan Africa studies have found 11% to 33% of patients with RB to have bilateral disease as seen in a study done in Republic of Côte d’Ivoire and the Democratic Republic of the Congo, which is in keeping with our study [11].

Leukocoria (69.2%, n=52) and proptosis (37.2%, n=25)) were the most common presenting symptoms. This was comparable to a study done at Kenyatta Hospital in Kenya at 71% and 37%, respectively [8]. Moreover, leukocoria was the most common presenting sign in Republic of Côte d’Ivoire and the Democratic Republic of the Congo [11,12]. However, this is different from data in middle-income to upper-income countries who present with leukocoria and strabismus as the most common signs, such as in Egypt and in the UK [13]. This is because symptoms, such as proptosis symptoms, are signs of advanced RB and present when there is most likely an orbital extension.

Intraocular tumours (stages pT1-3) constituted 73.1% (n=57), while extraocular tumours (stage pT4) were 26.9% (n=21). This was comparable to a study done in India that found intraocular tumours and extraocular tumours to be 72.3% and 27.7%, respectively [9]. PT4 was our second most common stage, which was consistent with the findings of a study done in India that showed pT1 of 48.1% and pT4 of 26% [14]. However, this is lower than the percentage found in a study done at REC, Uganda, that showed that almost half the tumours were extraocular (46%). This is due to the introduction of an effective safe chemotherapy regimen in Uganda, which presumably reduced the progression of disease to advanced stages [6].

An endophytic pattern was seen in 71.8% (n=56), an exophytic pattern was seen in 17.9% (n=14) and a mixed pattern in 10.3% (n=8). The incidence of growth patterns varies widely with the endophytic and mixed types being more predominant. This difference could be attributed to a difference in the biologic nature of these tumors [14]. 

Choroidal invasion was seen in 29.5% (n=23), which is comparable to that of Shields et al. (1993) of 23%. Although the incidence of choroidal invasion varies greatly in various reported series, ranging from 15.2% to 62%, it is lower than findings in other developing countries, such as in India with 47.4% [15]. This has been attributed to the limited peripheral calottes that were taken during the sectioning. However, massive choroidal invasion was seen in 12.8% cases, which is comparable to the 18% seen in Jordan, but it was still lower than that from other studies, such as in India at 24.6% because of the limited sectioning [15].

Reports indicate that 24% to 45% of eyes have a degree of ON invasion. ON invasion was seen in 38.5% (n=30), which was comparable with those in the study in America (38.7%) and India (32%) [16]. However, it was higher than that in a much earlier study in the USA, probably reflecting the advanced stage of the tumours in our study [17]. Retrolaminar ON invasion was seen in 3.9% (n=3) cases, which is comparable to that in Shields et al. (1994) of 5.5%; however, studies from developing countries have shown a higher percentage, such as Gupta et al. (2009) with 17% and Eagle (2009) with 10.4% [18,19]. Invasion of the resected margin of the ON was seen in 16.7% (n=13) cases. This was comparable to other studies in the developing world, but this is higher than that in developed countries, such as in the USA with 1% [17].

Scleral invasion was seen in 7.7% (n=6) cases, which was in keeping with other studies, such as that in Argentina with 8.8% and Pakistan with 7% [17]. Orbital extension was seen in 16.7% (n=13) cases, which is comparable to 18% in an Indian study [20]. This is due to the advanced stages of the tumours in developing countries. Invasion of the iris was seen in 16.7% (n=13) cases, which is in keeping with a study done in India with 10.7% [15]. The higher incidence of these risk factors in developing world might be related to later presentation (more advanced stage) in relation to the lower socioeconomic status and the delay in seeking and getting treatment [7].

Tumour differentiation is highly variable between different reports from the developing world. Many tumours showed a higher incidence of poorly differentiated (up to 80%) compared to well-differentiated tumours, which was comparable to our study of 65% (n=51) probably reflecting the late age of presentation of undifferentiated tumours [15].

Generally, necrosis was seen in 71.8% (n=56) cases, which was higher than in most studies because of the fact that most cases had undergone chemoreduction before enucleation in our study as compared to other studies that examined primarily enucleated eyes; however, extensive necrosis seen is 33.3% (n=26) was comparable to 31% of an Indian study [15].

Calcification in RB is a frequent histologic finding with a reported frequency between 40% and 95%, although the subject has not been studied in depth. Our study found calcification in 41% (n=32) cases, which is similar to the 48% in Malaysia, but this is lower than that seen in Israel at 84% [21].

Two-year survival was estimated to be 61.5% (n=48), and this was comparable to a study done in Taiwan at 64.4% [22]. This is higher than those of surrounding countries, such as in Kenya with 22.6% and in Tanzania with 23%, and this difference is assumed to be due to the development of an effective safe chemotherapy regimen in Uganda [6,8]. However, this is lower than those in developed countries, such as the UK where the survival rate is estimated at 95%. The poorer survival in low- and middle-income countries (LMICs) is attributed to a combination of many factors, including diagnostic delays resulting in advanced stage of disease at presentation, lack of availability of chemotherapeutic agents, cost of treatment leading to abandonment of care and limited access to surgery and radiotherapy [8].

Age was not found to be a predictor in our study as it is in many studies, but studies in India and Singapore have shown that age less than 24 months is a significant predictor, with children being able to survive most likely because children who present at a younger age may have tumours diagnosed at earlier stages of the disease [15].

Our study showed that sex had a significant influence on survival (RR 1.4), with females having a 1.4-fold chance of survival compared to males. Although most studies have not shown any difference in survival between males and females with RB, many studies have shown that females have a better cancer survival than males [23]. Although environmental and hormonal factors have been implicated in adulthood cancer, genetics have been thought to be the most common cause for this difference in childhood cancers as evidenced in some studies [24].

Most studies have shown that people with leukocoria have a better survival (RR 1.1), with our study showing that these people are almost 1.1 times more likely to be alive at two years than those without leukocoria. This is because leukocoria can easily be seen as an abnormal sign, and hence patients will present when the tumor is less advanced [8]. Most studies from developing countries, such as in Kenya, have shown that proptosis is associated with a poor survival as it is a sign of more advanced diseases, and this was consistent with this study that showed a significant association; however, this was not included in the final model as it would not achieve convergency at a multivariate analysis [8].

Cataract was a significant predictor at the univariate analysis but lost significance at the multivariate analysis. Cataract was a significant factor on the univariate analysis, and this is in keeping with some studies in India and the USA, which show that orbital cellulitis, phthisis bulbi, staphyloma and cataract are clinical predictors of high-risk pathology [15,25].

The rate of survival in patients with ON invasion depends on the degree of ON invasion. Survival rates increase as the degree of invasion reduces, and this was evidenced at the univariate analysis, where absent ON invasion, prelaminar ON and intralaminar ON invasion was significant [14]. However, at the multivariate analysis, there was statistical significance for only intralaminar ON probably due to fewer numbers.

Scleral invasion has been shown to be an independent factor of survival in most studies; however, this study did not provide an RR as there was no one in the alive group [14,26]. Choroidal invasion was seen to be significant in the univariate analysis but could not be included in the multivariate model because it could not allow for convergence, although mortality is higher in those with massive choroidal invasion.

Survival in patients with anterior segment invasion has been shown to be low, but our analysis could not produce RRs as there was no one in the exposure group, although mortality associated with these patients was very high.

Our study showed that orbital extension was significantly predictive of survival. Patients without orbital extension are able to survive seven times better than those with orbital extension. Orbital extension is a major cause of death in children with RB in developing countries, with mortalities of up to 100%. The presence of orbital invasion was associated with a 10-27 times higher risk of systemic metastasis as compared to cases without orbital invasion. This is in agreement with studies done in the USA and India [21].

Necrosis was a significant factor at the univariate analysis, although it lost significance at the multivariate analysis. Most studies including ours have not found necrosis to be associated with survival; however, a study done in the USA showed that patients with extensive necrosis are associated with high-risk pathology and mortality [27]. Our study did not show that differentiation is a predictive factor as it is in many other studies, but a study in Jordan has shown that poorly differentiated tumors are associated with more advanced tumor pathology [28]. Growth type has not been associated with survival although a study in Jordan illustrated that the mixed type independently affects survival as it would be found in advanced tumors [29]. Calcification was not shown to be a significant factor of survival, nevertheless no studies have been done to see its impact on survival.

Strengths and limitations

To the best of our knowledge, this was the first study in Uganda to extensively study the histopathological features of RB. 

Our study followed a retrospective study design that could have potentially limited access to some clinical information or outcome. The smaller sample size used in this study limits the generalizability of these results, and therefore we recommend studies using larger sample sizes. The majority of the histopathologic traits could not be compared to prior research conducted in the area.

## Conclusions

Leukocoria and proptosis are the most common clinical signs of RB. The dominant pathologic stage is stage 1, although late presentation (stage 4) is also common. Survival is still low, but it is higher than neighboring countries. Leukocoria, optic nerve invasion, orbital extension and gender were the significant factors predictive of survival in patients at Mbarara Regional Referral Hospital. Sensitization of health workers and community on the identification and referral of any child with leukocoria to improve histopathology reporting in order and to identify patients at risk is highly recommended.
